# The Hippo Pathway in Kidney Development

**DOI:** 10.3390/encyclopedia5010015

**Published:** 2025-01-26

**Authors:** Caroline M. Lara, Toni Castro Torres, Usaid Mazhar, Dorrian G. Cohen, Rebecca A. Wingert

**Affiliations:** Department of Biological Sciences, University of Notre Dame, Notre Dame, IN 46556, USA

**Keywords:** Hippo pathway, kidney development, YAP, TAZ, nephron

## Abstract

The kidney, a complex organ crucial for a multitude of homeostatic functions, including the maintenance of fluid and electrolyte balance, removal of various metabolic waste products, and hormone production, undergoes intricate developmental processes to form functional nephron units. Understanding the mechanisms behind kidney development is paramount for elucidating the etiology of renal congenital disabilities and acquired diseases. The Hippo pathway is known for its involvement in various cellular functions, including cell fate determination and differentiation, and is a critical player in renal organogenesis. Here, we discuss research findings about the roles that Hippo signaling plays in kidney formation.

## 1. Introduction

Kidney organogenesis is a complex process involving the stepwise formation and degradation of several increasingly sophisticated renal structures [1,2]. Ongoing advances in elucidating the molecular events that direct kidney formation are crucial for continuing to illuminate the underpinnings of birth defects that are known collectively as congenital anomalies of the kidney and urinary tract (CAKUT) [1,2]. Further, mechanisms of kidney development are relevant to the pathogenesis of renal afflictions ranging from acute to chronic disease conditions. To date, the Hippo signaling pathway has been extensively characterized for its roles in many important events during ontogeny, such as directing organ size, cell fate, cell proliferation, cell differentiation, and survival [3]. Two critical components of the Hippo pathway are the downstream effectors known as Yes-associated protein 1 (YAP) and its paralog WW domain-containing transcription regulator 1 (WWTR1), commonly referred to as TAZ based on its alternative name, Transcriptional coactivator with PDZ-binding motif [3]. YAP and TAZ are targeted for phosphorylation by the upstream Hippo kinases, leading to their nuclear exclusion and degradation [3]. Conversely, at times when Hippo signaling is low or absent, YAP and TAZ can translocate to the nucleus and interact with binding partners to facilitate the transcription of gene targets. At present, it is not fully understood how the Hippo pathway kinases and their various downstream effectors influence the events of kidney development, but that gap is steadily closing. This concise review summarizes the current knowledge of the Hippo pathway in the kidney during organogenesis, with a special focus on the roles of YAP and TAZ.

## 2. Kidney Development

**Figure 1.**
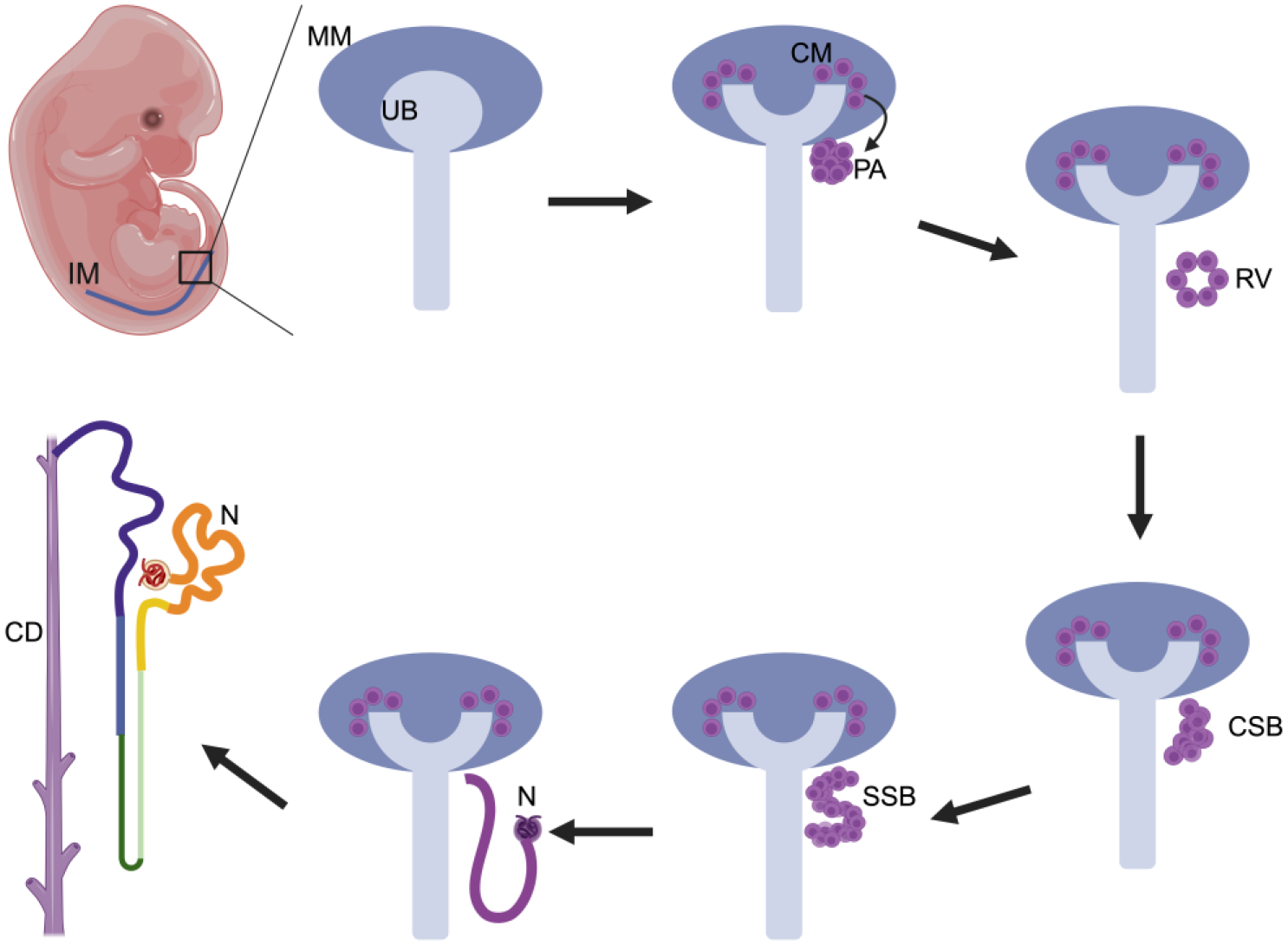
Development of the metanephric kidney. The intermediate mesoderm (IM) undergoes outgrowth to make the ureteric bud (UB) which is surrounded by metanephric mesenchyme (MM). MM closely associated with the UB, known as the cap mesenchyme (CM) is induced to form a pretubular aggregate (PA) which develops into a renal vesicle (RV). Growth of the RV is visualized first as a comma-shaped body (CSB), then an S-shaped body (SSB), then a rudimentary nephron (N). Each nephron undergoes differentiation to make a segmented tubule (depicted by colored regions) which attaches to the collecting duct (CD) system formed by the branching morphogenesis of the UB. Made with Biorender.com.

The kidney is a vital organ because of its essential role in fluid homeostasis. The kidney performs functions that accomplish waste excretion, regulation of osmolarity, maintenance of ion balance, management of acid-base levels, and the hormonal control of blood pressure and hematopoiesis. Mammalian kidney development involves a series of intricate events that drive progressive pattern formation of the intermediate mesoderm [1]. The mammalian kidney emerges in a multiplex fashion, involving three organ forms that develop and degrade during development, known as the pronephros, mesonephros, and metanephros [2]. Each kidney is comprised of basic structural and functional units known as nephrons which are comprised of a blood filter and a tubule that reabsorbs and secretes solutes. The pronephros and mesonephros typically consist of a small number of nephrons (<20) and exist for a relatively short period of time in embryogenesis. Creation of the architecturally sophisticated metanephros, which will generate thousands to millions of nephrons depending on the species, relies on dynamic interactions between the ureteric bud (UB) and a population of surrounding mesenchymal nephron progenitor cells (NPCs) (Figure 1) [3–5]. The UB undergoes branching morphogenesis in response to signaling from the mesenchymal NPCs, growing to form the collecting duct system which serves to gather and drain urine. In turn, reciprocal signaling from the UB branch points induces groups of adjacent NPCs, known as the cap mesenchyme (CM) cells, to undergo a mesenchymal-to-epithelial transition (MET) and form a pretubular aggregate (PA). Each PA is a precursor to a nephron, first becoming a structure known as the renal vesicle (RV). The RV proliferates, elongating in shape to form a comma-shaped and then an S-shaped body that ultimately fuses with the distal ureteric bud. Over time, the S-shaped body will differentiate into a mature nephron with a series of distinct proximal, intermediate, and distal functional segments, and becomes vascularized by endothelial cells that contribute to making the blood filter and peritubular capillary networks [4–6].

Once formed, each nephron functions by allowing blood to flow through the afferent arteriole into the glomerulus, which is comprised of a ball of capillaries and encased by Bowman’s capsule. Filtration occurs as materials from the bloodstream move across the capillary fenestrated endothelial cells, through the surrounding basement membrane, and between spaces of the opposing renal podocytes [7]. After filtrate passes into the space of the Bowman’s capsule, it travels through the long nephron epithelial tubule where it will be selectively modified in transit by the sequence of segments that each performs discrete physiological tasks. These modifications involve the selective reabsorption and secretion of molecules such as glucose, amino acids, ions, and water, cumulatively fashioning urine and exquisitely balancing electrolyte and fluid levels in the body [8]. For example, the proximal tubule performs glucose reabsorption, the loop of Henle reabsorbs water and creates an osmotic gradient for urine concentration, and the distal tubule performs fine-tuning of salt levels [9–12].

## 3. Hippo Pathway

Initially discovered in the fly, *Drosophila melanogaster*, the Hippo pathway is highly conserved and essential across metazoans. Hippo pathway signal transduction involves a kinase cascade that regulates cellular growth and development across many organs by influencing cell proliferation and differentiation via the activities of the transcriptional coactivators YAP and TAZ [3,13]. For example, disruption of any one element of the Hippo pathway can compromise different aspects of kidney growth and development, and there is considerable variability depending on which pathway component is altered.

Several critical components of the Hippo pathway allow for the regulation of various downstream expressions of genes that mediate cell proliferation, apoptosis, and stem cell renewal (Figure 1) [14]. In mammals, the key components are structured in a cascade, such that mammalian STE20-like kinase 1/2 (MST1/2) (also known as STK4/3), protein Salvador homolog 1 (SAV1), MOB kinase activators 1A and 1B (MOB1A/B) (MOB1A/B), large tumor suppressor kinase 1/2 (LATS1/2), Yes-associated protein 1 (YAP), WW-domain-containing transcription regulator 1 (TAZ) (also known as WWTR1), and the transcriptional enhanced associated domain (TEAD) family are acted upon, respectively [15].

**Figure 2.**
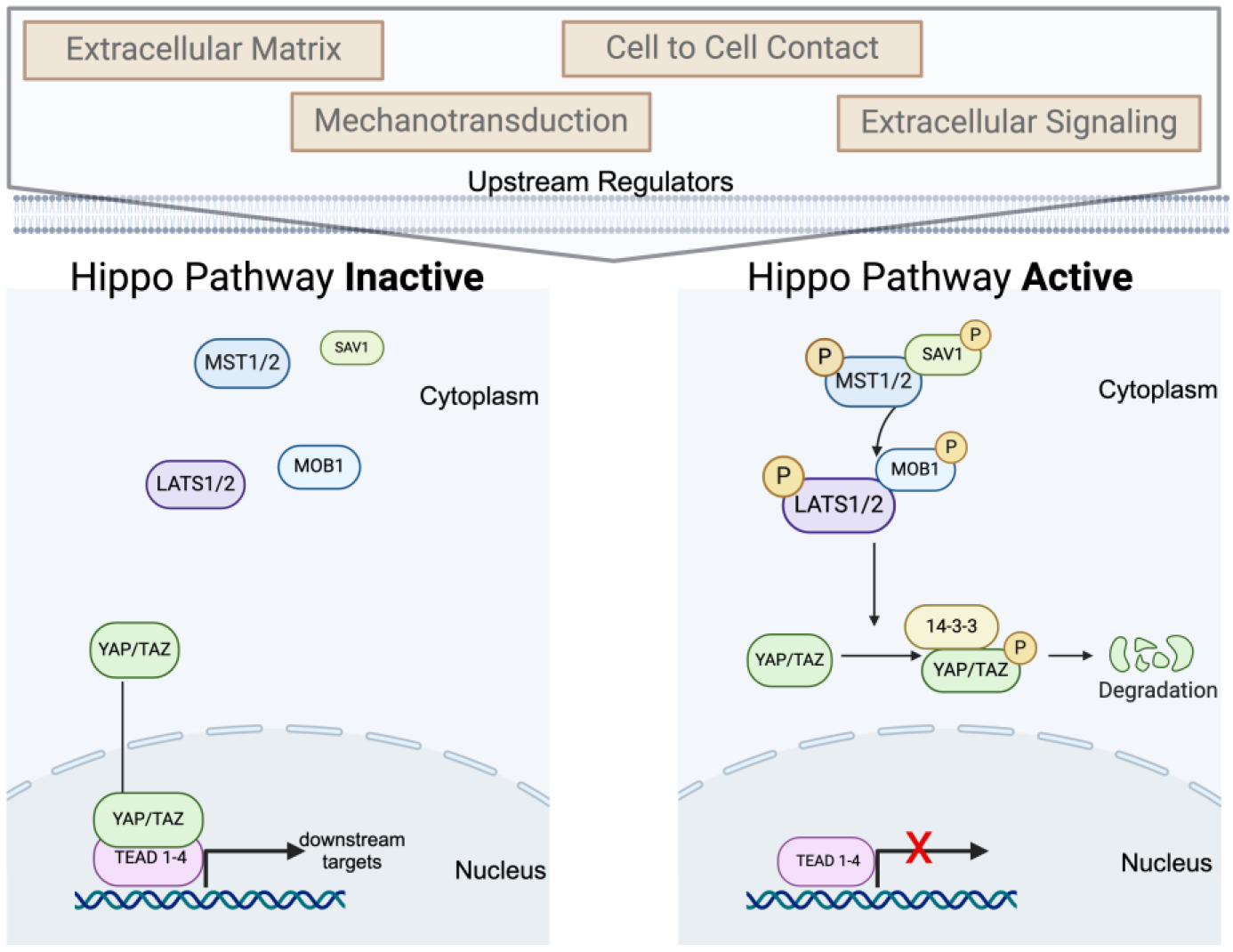
The Hippo signaling pathway. Members of this kinase cascade act in a sequential fashion beginning with mammalian STE20-like kinase 1/2 (MST1/2) (STK4/3), Salvador homolog 1 (SAV1), MOB kinase activators 1A and 1B (MOB1A/B), large tumor suppressor kinase 1/2 (LATS1/2), the Yes-associated protein 1 (YAP), and WW-domain-containing transcription regulator 1 (TAZ) (WWTR1). When YAP/TAZ are not phosphorylated, they translocate to the nucleus and bind several transcriptional enhanced associated domain (TEAD) coactivators to regulate the expression of target genes. Made with Biorender.com.

In general, for the activation of the Hippo pathway, MST1/2 and SAV1 phosphorylate LATS1/2 and its scaffold MOB1A/B [16]. Activation of LATS1/2 promotes phosphorylation of the transcriptional regulators YAP and TAZ, resulting in the sequester in the cytoplasm, excluding YAP and TAZ from the nucleus [3]. This phosphorylation additionally results in the eventual degradation of YAP and TAZ by the ubiquitin–proteasome system [17]. Therefore, the Hippo pathway has two states: “active” is when YAP/TAZ are phosphorylated in the cytoplasm, and “inactive” is when YAP/TAZ lack this modification and translocate to the nucleus where they can bind to TEAD factors and facilitate gene transcription of various targets (Figure 2). Various upstream stimuli modulate the Hippo pathway, including but not limited to mechanotransduction, metabolism, extracellular matrix, extracellular signaling, cell polarity, and cell–cell contact [18].

## 4. Expression of Hippo Pathway Components in the Kidney

The broad necessity of the Hippo pathway has made its roles in processes like kidney development and, specifically, nephrogenesis, an ongoing and important field of study. Expression of the various Hippo pathway components has been curated using several methods and compiled in an online database [19]. For example, in the 17-week human kidney cortex, the Hippo pathway components were found to be expressed in differing populations. MST1/2 (STK4/3) was found to be expressed in tubular precursors, endothelium, and immune cells. LATS1/2 was expressed in the nephron progenitors, interstitial progenitors, and tubular precursors. YAP was expressed in podocytes, nephron progenitors, and tubular precursors. Interestingly, TAZ was expressed in the endothelial cells and minimally in other populations. Further studies are needed to determine if the complete Hippo pathway is expressed in all renal cell types, and at what developmental times. However, these expression data provide useful insights that have been relevant to the design and interpretation of various genetic studies on the Hippo pathway in renal cell types, such as the effectors YAP and TAZ, e.g., [20–28].

## 5. Hippo Signaling in Glomerular Development and Podocyte Maintenance

The functions of the Hippo pathway in glomerular development have yet to be fully established. Most studies to date implicate Hippo signal transduction as important in the homeostasis and survival of podocytes, also known as glomerular visceral epithelial cells. Podocytes form a crucial component of the filtration apparatus in the nephron functional units of the kidney by ensuring proper selective filtration. Podocytes are unique epithelial cells enwrapping the glomerular capillaries, forming elaborate cell extensions termed foot processes that interdigitate to create filtration slits composing the kidney filtration capillaries, forming elaborate cell extensions termed foot processes that interdigitate to create filtration slits composing the kidney filtration barrier [26]. These slits are vital in the regulation of proteins in the blood and proper filtrates through the filtration barrier, preventing the escape of essential proteins while allowing waste products to be excreted in urine [27]. Their intricate structure and function make podocytes pivotal in preventing proteinuria and maintaining overall renal health.

Genetic depletion of Yap in the CM during metanephros development in the mouse was shown to dramatically disrupt the processes involved in the formation of the glomerulus. *Yap*^*CM*−/−^ mutant kidneys have a reduced number of glomeruli [23]. The few glomeruli that were detected displayed ultrastructural abnormalities. The capillary tuft that would normally have a tight encasement of podocytes displayed podocyte effacement [23]. These defects were linked to changes in the expression of genes that are responsible for directing cell fate and morphogenesis, as opposed to the proliferation or survival of nephron progenitors [23].

Several studies have demonstrated that alterations in YAP expression significantly alter podocyte homeostasis. As previously described, the activation of LATS1/2 is well known to negatively regulate the transcriptional activity of YAP and TAZ by promoting their cytoplasmic localization (Figure 2). In keeping with this signaling paradigm, the presence of a constitutively active LATS kinase in podocytes leads to the nuclear export of YAP [29]. Several studies have demonstrated that the nuclear localization of YAP is important for podocyte survival. For example, when the LATS activator WW and C2 domain containing 1 (WWC1, also known as KIBRA or “kidney brain” protein) is conditionally overexpressed, this results in YAP nuclear export and causes an increased level of podocyte apoptosis [29]. In studies where researchers cultured podocyte cells and treated them with dasatinib, a small molecule that acts as an inhibitor of several receptor tyrosine kinases that was previously shown to inhibit the nuclear localization of YAP, podocytes similarly displayed a loss of nuclear YAP and enhanced apoptosis [28]. The loss of nuclear YAP was associated with decreased transcription of several anti-apoptotic genes that included B cell lymphoma 2 (Bcl2) [28]. These in vitro studies suggest that sustaining the proper level of LATS expression is required for the appropriate maintenance of podocytes. Further, the use of a YAP inhibitor drug, verteporfin, induced podocyte apoptosis through nuclear exclusion of YAP [30]. Therefore, sustaining homeostatic LATS expression and the nuclear location of YAP are requisite for the appropriate development of podocytes.

Podocyte-specific depletion of YAP or its nuclear exclusion in podocytes has been linked to the onset of focal segmental glomerulosclerosis (FSGS) [17,31]. While researchers found that podocyte-specific *Yap* knockout mice did not display nephron defects at birth, the animals developed proteinuria by 12 weeks of age and several pathologies including glomerular scar formation, tubular injury, interstitial fibrosis, and interstitial inflammation [31]. Podocyte apoptosis was detected as early as 7.5 weeks, continued at 12 weeks, and led to marked podocyte depletion [31]. Further, analysis of human FSGS samples revealed reduced glomerular expression [31]. A more recent examination of human FSGS samples detected an elevation in cytoplasmic YAP in podocytes and—consistent with this—detected elevated levels of phosphorylated YAP in human FSGS as well [17]. Using an adriamycin-induced FSGS mouse model, another team of researchers found that there was a gradual nuclear exclusion of YAP in podocytes over time that was coincident with elevated phosphorylation of YAP as well as elevated podocyte apoptosis [17]. Taken together with the analysis of *Yap*^*CM*−/−^ mutant kidneys, these results indicate that YAP has an integral role in ensuring healthy podocyte development and preventing the onset of renal disease.

The paralog downstream effector of the Hippo pathway, TAZ, is also essential in podocyte homeostasis. The consequences of TAZ abrogation on podocyte development were revealed using a podocyte-specific knockout model, the *Taz*^*podKO*^ mouse [24]. While approximately 40% of *Taz*^*podKO*^ mice developed mild proteinuria beginning at 4 weeks of age, this was pronounced in all *Taz*^*podKO*^ mice by 5–6 weeks, followed by progressive FSGS at 9 weeks of age [24]. In tandem, the *Taz*^*podKO*^ animals displayed reductions in podocyte numbers, as well as increased podocyte apoptosis [24]. Notably, molecular analysis of the glomerular lysates from *Taz*^*podKO*^ kidneys revealed elevated levels of YAP in the nuclear and cytosolic fractions, suggesting the existence of an endogenous regulatory response to offset the loss of TAZ. However, the elevations in YAP were not sufficient to compensate for TAZ depletion given the development of proteinuria and FSGS in this model. Mice who survived Taz gene deficiency during the perinatal stage developed bilateral kidney cysts and pulmonary emphysema. These phenotypes are potentially observed because TAZ predominantly governs the genes needed for cell migration and extracellular matrix remodeling [32].

## 6. Hippo Signaling in Nephron Tubule Development

Elegant genetic studies have explored the roles of the Hippo pathway components in nephron tubule development, and have identified several essential players. Beginning at the first kinase involved in Hippo pathway activation, it has been shown that MST1/2 expression does not influence embryonic kidney development but affects the overall health of the adult kidney over time [13]. Following Ksp-Cre-mediated *Mst1/2* deletion in the tubular cells of the mouse kidney, there was no significant change in the size of the kidney at two days or two weeks of age, but kidney size increased after four weeks in *Mst1/2* double knock-out mice [13]. These results suggested that MST1/2 are necessary to prevent kidney overgrowth. Consistent with this, the proliferation of tubular cells was increased in *Mst1/2* double-knockout mice [13]. There was an increase in YAP phosphorylation and elevated nuclear YAP in nephrons of *Mst1/2* double knockout mice as well, but TAZ localization and activity were unchanged compared to controls [13]. This indicates that YAP and TAZ are differentially regulated in nephron tubular cells. Interestingly, the expression of MST1, MST2, and YAP was not detected in proximal tubules [13]. This intriguing observation suggests that the function of these genes is not necessarily shared across nephron tubule segment populations. Over time, the *Mst1/2* double knockout mice displayed progressive tubular damage that was associated with damage to mitochondria, as well as interstitial inflammation and increased renal fibrosis [13]. Collectively, these phenotypes were mostly attributed to YAP as they were rescued by tubule-specific *Yap* depletion [13].

Similarly, with the kidney-specific knockout of *Sav1* in a murine model, histological analyses showed increases in the proliferation of epithelial cells in renal tubules [33]. Kai et al. showed that *Sav1* knockout mice have renal tubular abnormalities, such as irregular nuclei, multilayered epithelium, and renal cysts. These are indicators of the importance of *Sav1* in proper kidney development. A conditional knockout of *Sav1* produces genetic and phenotypic alterations representative of renal fibrosis. It is crucial to note that reduced YAP phosphorylation was not observed as a consequence of *Sav1* genetic deletion [34]. This indicates that *Sav1* may not function to promote the degradation of YAP through the canonical Hippo pathway. Instead, *Sav1* loss may direct a molecular cascade that operates independently of the Hippo pathway to promote activation of *Yap* and pro-fibrotic genes, thereby inducing fibrosis [34].

Conditional knockout of Lats1/2 in the mouse kidney CM was found to cause the development of dramatically undersized kidneys that displayed a loss of nephrons, where neither glomeruli nor tubules formed in the kidney [3]. The loss of *Lats1/2* caused nephron progenitors to accumulate within the interstitium [3]. These cells lacked epithelial characteristics and instead displayed fibrotic characteristics such as the expression of α-smooth muscle actin, suggesting they were converted into myofibroblasts in the kidney [3]. Elevated YAP and TAZ activity were associated with these changes, and genetic knockout of either *Yap* or *Taz* rescued nephrogenesis [3]. These studies revealed the crucial necessity of *Lats1/2* to regulate YAP and TAZ in NPCs to ensure the proper differentiation of nephron progenitor cells which involves a MET.

In murine models, genetic deletion of *Yap* and *Taz* deletion has revealed that YAP and TAZ have distinct roles in the CM nephron progenitor cells during mammalian kidney development [20–23]. Conditional murine knockout of *Yap* in the CM cells (*Yap*^*CM*−/−^) led to early postnatal mortality, where pups survived to approximately 48 h postbirth [23]. Examination of the *Yap*^*CM*−/−^ kidneys revealed numerous anatomical anomalies [23]. The *Yap*^*CM*−/−^ kidneys were hypoplastic and the animals had empty bladders, consistent with a reduced or absent ability to generate urine [23]. Further, *Yap*^*CM*−/−^ kidneys possessed a reduced nephrogenic zone along with a reduction in the number of nephrons based on a reduction in detectable proximal tubule segments and glomeruli, along with defects in the formation of the distal tubule and loop of Henle [23].

The conditional knockout of *Taz* in the CM does not have the identical phenotypes as *Yap* [23]. *Taz* genetic deletion led to cyst formation within the tubules and was later found to cause defects in urine concentration [35]. Interestingly, double mutants deficient in *Yap* and *Taz* in the CM do not exacerbate the phenotypes [23]. Though YAP and TAZ share 45% amino acid identity and 60% protein sequence similarity, and their roles within the Hippo pathway are commonly thought of as duplicative, these studies have clearly demonstrated that each effector has unique roles in ensuring distinct aspects of successful kidney development [23].

Interestingly, in the zebrafish *Danio rerio*, *yap* and *taz* are necessary for pronephros tubule development. The zebrafish pronephros is a renal structure that is composed of two nephrons that possess a segmental organization that is conserved with mammals [36,37]. Specifically, it has been shown that *taz* is essential for the proper anterior–posterior patterning of the pronephric progenitor field [36]. Upon *taz* knockdown in zebrafish embryos, there was a decrease in the monociliated cells in the proximal straight tubule and distal early tubule segment, along with a proximalization of the pronephric tubule. Zhang et al. attributed these phenotypes to the necessity of *taz* interacting with the morphogen retinoic acid [38]. Zebrafish with *yap* deficiencies display defective pronephric development and cyst formation [39]. Utilizing a morpholino oligomer to knockdown expression of *yap*, He et al. showed that *yap* morphants have discontinuous and dilated pronephric duct, glomerular cysts, and cell migration defects [39]. These changes were not attributed to cell fate transitions or disruptions of cell proliferation [39]. Taken together, these studies demonstrate that Yap and Taz ensure healthy tubular development in the pronephros.

## 7. Conclusions and Prospects

There is increasing evidence that Hippo signaling serves a number of essential roles in the emergence of the vertebrate kidney. From the kinases to the downstream effectors, each has a significant role in the development or maintenance of the nephrons to ensure their functionality. The Hippo pathway has been implicated in various diseases, can induce tumors, and is expressed in carcinomas in the lung, ovary, and liver [40]. Recent studies have implicated the Hippo pathway in various kidney pathologies as well, specifically diabetic nephropathy, renal fibrosis, acute kidney injury (AKI), and polycystic disease [41,42]. These conditions all have phenotypes that include those caused by the depletion or overexpression of the Hippo pathway. For example, mild or moderate AKI has been associated with transient activation of YAP/TAZ and correlates with positive recovery of renal tubule epithelial cells [43]. In contrast, persistent activation of YAP/TAZ has been observed following severe or repeated AKI and is associated with maladaptive responses, such as tubulointerstitial fibrosis, that lead to the progression of chronic kidney disease (CKD) [43]. Further studies of the Hippo pathway will be useful in further elucidating the pathophysiology of these conditions. The Hippo pathway has been shown to crosstalk with various other signal transduction cascades, such as Wnt signaling, Notch signaling, and TGF-β, especially when involved in disease [41,42]. Therefore, further work is needed to advance drug development, treatment, and understanding of renal pathological states resulting from alterations in the Hippo pathway. Researchers have examined the use of kinase inhibitors against MST1/2 or LATS1/2 to modulate YAP/TAZ activity, as well as agents that directly target YAP/TAZ [44]. Furthermore, there may be promise in targeting the molecules that act downstream of YAP/TAZ [44]. Determining whether such agents can be efficacious in renal afflictions may realize many new opportunities for innovative therapeutics in the years to come.
